# *Chiococca grandiflora* (Rubiaceae), a new species from Northern Mexico

**DOI:** 10.3897/phytokeys.98.25170

**Published:** 2018-05-02

**Authors:** David H. Lorence, Thomas R. Van Devender, George M. Ferguson

**Affiliations:** 1 National Tropical Botanical Garden, 3530 Papalina Road, Kalaheo, HI 96741, USA; 2 GreaterGood.org, 6262 N. Swan Rd., Suite 150, Tucson, AZ 85718, USA; 3 University of Arizona Herbarium (ARIZ) 1140 E. South Campus Dr. Tucson, AZ 85721-0036, USA

**Keywords:** *Chiococca*, conservation, Mexico, Rubiaceae, Sinaloa, Sonora

## Abstract

The new species *Chiococca grandiflora* Lorence & T.Van Devender from Sinaloa and Sonora, Mexico differs from its congeners by its larger, showy white flowers in compact cymes of 3–9, and infundibuliform corollas 16–20 mm long with tubes 13–17 mm long and lobes 3–3.5 mm long. Its distribution, habitat, and relationships are outlined. The conservation status for this species is estimated to be Endangered (EN) based on IUCN Red List Criteria.

## Abstract (es)

La nueva especie, *Chiococca grandiflora* Lorence & T.Van Devender, de Sinaloa y Sonora, México, difiere de sus congenéricas por sus grandes y vistosas flores blancas, en cimas compactas de 3–9 flores, y corolas infundibuliformes de 16–20 mm de longitud, con tubos de 13–17 mm y lóbulos de 3–3.5 mm de longitud. Se presentan datos de distribución, hábitat y relaciones. De acuerdo con los criterios de la Lista Roja de la IUCN, se estima que el estado de conservación de esta especie debe ser como En Peligro (EN, por sus siglas en Inglés).

## Introduction

*Chiococca* P. Browne (Rubiaceae, Cinchonoideae: Chiococceae) is a taxonomically complex genus of about 25 species of Neotropical shrubs and small trees. The type species is *Chiococca alba* (L.) Hitchc., a widespread and morphologically extremely variable shrub or small tree ranging from northern Mexico and the Caribbean south to Argentina. Borhidi (2006) recognized 13 species for Mexico in his *Rubiáceas de México*. The genus was revised for the 10 species occurring in the Mesoamerica region by Lorence and Taylor (2012). Several additional species occur in the Caribbean and South America.

Traditionally within Rubiaceae *Chiococca* has been placed in subfamily Cinchonoideae, tribe Chiococceae which is strongly supported by recent molecular evidence (Manns and Bremer 2010). This genus is distinctive but its species are often difficult to key out, however, especially since mature flowers and fruits rarely occur together on the same collection, but these both are often essential for identification. Certain taxonomically useful vegetative and floral characters tend to be subtle and overlap or intergrade in certain species, often making identification a challenge.

A distinctive new species of *Chiococca* with large white flowers was discovered and collected by Sally Walker in 1970 in Sinaloa, Mexico, two miles west of El Palmito near the Durango-Sinaloa border, and also at 19 km west of El Palmito (Figure 3). A third specimen was collected in 1978 near El Palmito, Sinaloa by Tim Walker. Collections of the same species were made in 1992 by Paul S. Martin of the University of Arizona while conducting field work in Sonora at Sierra Saguaribo near Tepopa for the revision of Howard Scott Gentry’s *Rio Mayo Plants* (Gentry 1942; Martin et al. 1998). This plant was reported as *Chiococca* sp. nov. by Martin et al. (1998) in the book *Gentry’s Rio Mayo Plants*. Comparison with all other species of the genus revealed it represents a new species described below.

## Taxonomy

### Chiococca grandiflora

Taxon classificationPlantaeGentianalesRubiaceae

Lorence & T.Van Devender sp. nov.

urn:lsid:ipni.org:names:60476299-2

#### Type.

**Figure 1.**
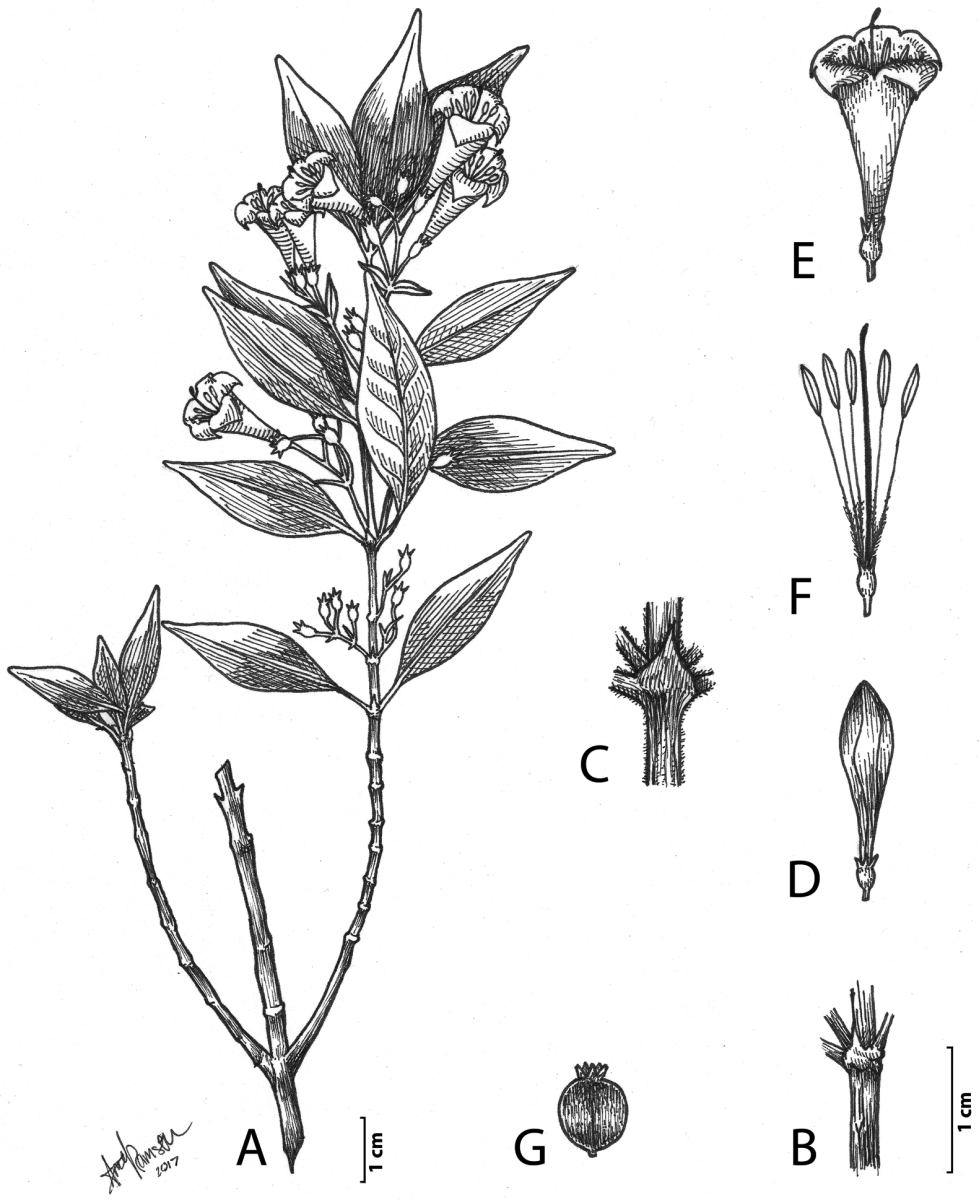
*Chiococca grandiflora* Lorence & T.Van Devender. **A** flowering branch **B** node showing stipule and petiole bases, glabrous form **C** node showing stipule and petiole bases, pubescent form **D** flower in bud **E** flower at anthesis **F** flower with corolla removed showing stamens and pistil **G** immature fruit. **A, B, D–F** based on P. S. Martin, P. Comtois, C. Lindquist, S. A. Meyer, B. Risner, & D. A. Yetman s.n. sub P. Jenkins 92-135 (ARIZ-383348) **C** based on S. Walker s.n. (UTC-00263027) **G** based on T. Walker s.n. (ARIZ-212520).

**Figure 2.**
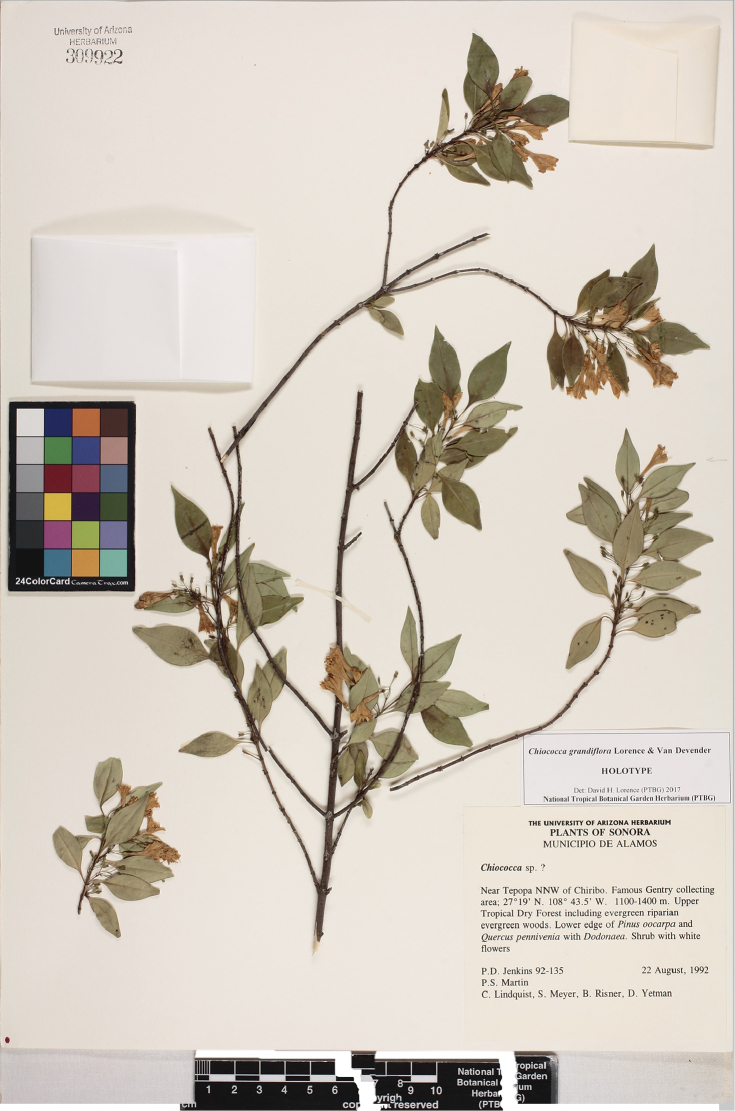
*Chiococca grandiflora* Lorence & T.Van Devender. Holotype collection, P. S. Martin, P. Comtois, C. Lindquist, S. A. Meyer, B. Risner, & D. A. Yetman s.n. sub P. Jenkins 92-135 (ARIZ-309922).

MEXICO. Sonora, Municipio Alamos. Near Tepopa NNW of Chiribo, 27°19'N, 108°43.5'W, 1100–1400 m, 22 August 1992 (fl), *P. S. Martin, P. Comtois, C. Lindquist, S. A. Meyer, B. Risner, & D. A. Yetman s.n. sub P. Jenkins 92-135* (Holotype: ARIZ-309922!; Isotypes ARIZ-383348!, PTBG-105887!) (Figures 1, 2). [Note: The ARIZ isotype specimen label says “Abandoned orchard of Tepopa” at 1100–1400 m elevation.]

*Chiococca grandiflora* differs from other members of the genus by its relatively larger, showy white flowers in compact cymes of 3–9, and its infundibuliform corollas 16–20 mm long with tubes 13–17 mm long and lobes 3–3.5 mm long.

Shrubs to 3 m tall, branches erect-ascendent, branchlets glabrous or sometimes persistently short hirtellous with white trichomes 0.05–0.1 mm long. Leaves of a pair equal, petiolate; blades ovate, 1.8–6.5 cm long, 0.8–2 cm wide, glabrous or sometimes minutely hirtellous on both surfaces, stiffly chartaceous to subcoriaceous, apex acute or acuminate to short-acuminate, base cuneate, decurrent, margin weakly revolute, secondary veins 3–5 on each side, brochidodromous, inconspicuous on both surfaces; petioles 3–4 mm long, glabrous or sometimes short-hirtellous; stipules triangular, 2–3 mm long, acute and aristate, the awn subulate, 1.5–2.5 mm long, the base 1–1.5 mm long, thick and persistent, externally glabrous or sometimes short hirtellous, internally with colleters. Inflorescences axillary, 3–4 cm long, 2–2.5 cm wide, racemose or sometimes with a pair of basal secondary branches, glabrous or sometimes short-hirtellous, rachis 5–12 mm long, floral bracts 1–3 mm long, triangular to linear-subulate, acute, glabrous or the margins short-hirtellous; flowers 3–9, pendulous, hypanthium 1.5–2.2 mm long, obovoid-ellipsoid, laterally compressed, glabrous or sometimes short-hirtellous; calyx limb below the lobes 0.5–0.7 mm long, tubular, short-hirtellous or only the margins hirtellous, calyx lobes 5, 0.4–1.0 mm long, triangular-subulate, acute, recurved, glabrous or the margins hirtellous, glabrous within; corolla infundibuliform, 16–20 mm long, white, externally sparsely hirtellous, internally glabrous, tube 13–17 mm long, 6–11 mm wide at throat, lobes 5, triangular, 3–3.5 mm long and wide, recurved at anthesis; stamens with tips exserted for 1–2 mm, filaments 8–12 mm long, minutely strigillose, attached near base of tube, anthers linear, 3–3.2 mm long; style exserted for 2–3 mm, 16–18 mm long, glabrous, tip swollen for ca. 1 mm, with stigmatic portion decurrent for ca. 5 mm laterally. Fruits drupaceous, spongy at maturity, 5–5.5 mm in diameter, broadly ellipsoid to subglobose, compressed, white, glabrous. Seeds not seen.

#### Additional specimens examined (paratypes).

MEXICO. Sinaloa. Municipio Concordia. Rock Slide, El Palmito, Sinaloa, elev. 7000 ft, 18 March 1978 (fr), *T. Walker s.n.* (ARIZ-212520); Municipio Concordia, 2 miles W of El Palmito, Sinaloa, 7000 ft, pine oak, 1 September 1970 (fl), *S. Walker s.n.* (UTC-00263027; ARIZ-181630); Municipio Concordia, 19 km W of El Palmito, Sinaloa, in Sinaloa, 6200 ft., 1 September 1970 (fl), *S. Walker 70,043* (K, loan # H2017/00697).

**Figure 3.**
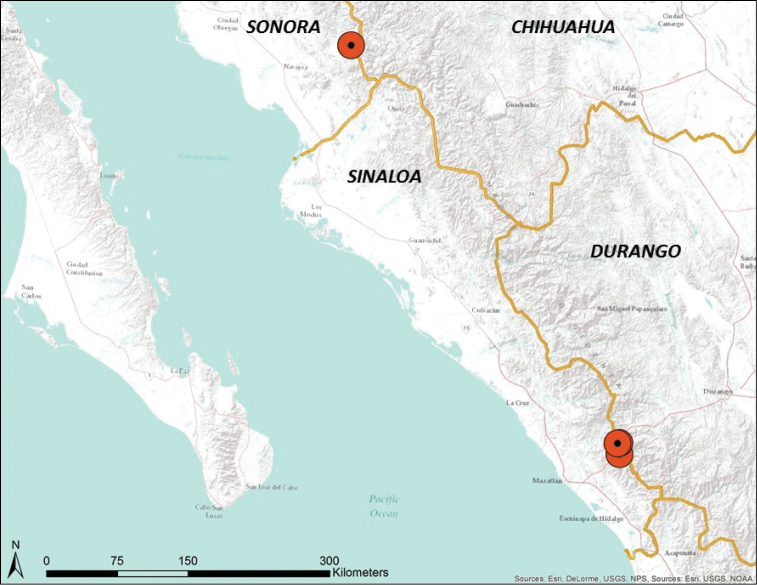
Distribution of *Chiococca grandiflora* in western Mexico.

#### Discussion.

**Figure 4.**
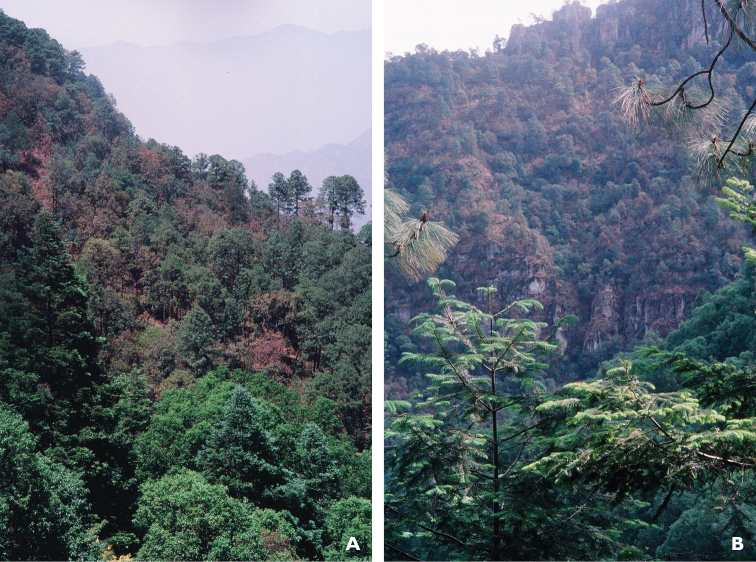
Habitat of *Chiococca grandiflora* near paratype location ca. 2 miles NW of El Palmito, Sinaloa, Mexico showing deep barrancas with pine-oak forest transitional to tropical deciduous forest.

**Figure 5.**
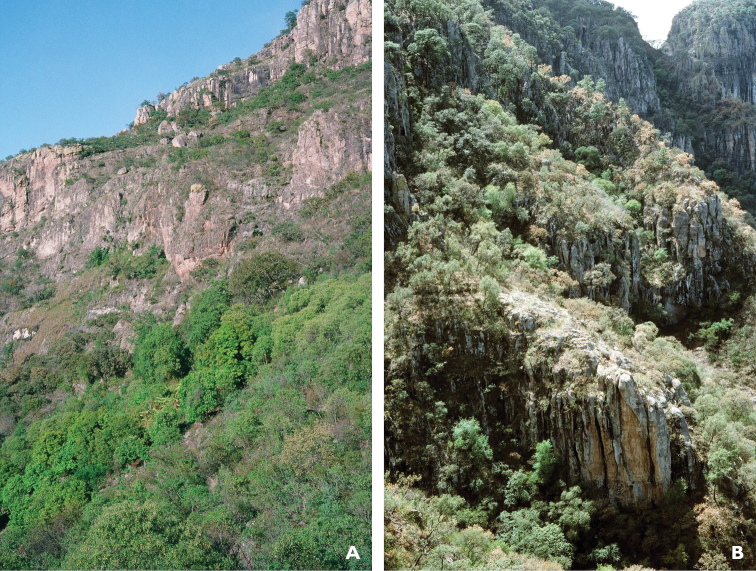
Habitat of *Chiococca grandiflora* in vicinity of holotype location near Tepopa, Sonora, at ecotone of upper tropical deciduous forest including riparian evergreen forest, and lower edge of pine-oak forest. **5a** showing abandoned orchard with fruit trees including bananas near center.

*Chiococca grandiflora* displays some variation in vegetative and floral pubescence. We were at first inclined to select as holotype the most amply floriferous specimen, from Sinaloa 2 miles W of El Palmito collected by Sally Walker *s.n.* on 1 September 1970 (UTC-00263027), since there is a putative duplicate of this collection (ARIZ-181630; gathered at the same locality on the same date). However, the UTC specimen has uniformly short-hirtellous twigs, leaves, and inflorescences with white trichomes 0.05–0.1 mm long (Figure 1C), compared with the ARIZ specimen which is essentially glabrous except for the calyx lobes having ciliate margins. Although the collections presumably were gathered from the same population, they were almost certainly taken from different individuals. In any case, it suggests that pubescence is variable in this species, and this variation is encompassed by the description above. The other Sinaloa specimens, *T. Walker s.n* (Rock Slide, El Palmito, ARIZ-212520) and *S. Walker* 70,043 (19 km W of El Palmito, K), and the type collection from Sonora are all similarly glabrous. Field studies would help elucidate this variation. However, localities of the known populations in Sinaloa are remote and those in Sonora are rugged and relatively difficult to access (Figures 4, 5). The variation in pubescence documented here is not unusual for a species of *Chiococca*.

Regarding the type collection, Paul Martin was most likely the actual collector, although Martin did not assign numbers to his collections, only dates. Consequently, Phil Jenkins catalogued the specimen and assigned it his number 92-135. The paratype collections by Sally Walker (and Tim Walker) are from near the Durango border and were originally designated on the specimen label as either “west of El Palmito, Durango in Sinaloa” or “near El Palmito, in Durango” as the state line was not exactly known. El Palmito and immediate surroundings are in Sinaloa, however, and the state line is farther east than they realized.

#### Relationships.

The relationships of *Chiococca grandiflora* within its genus are unclear, along with those of all the other species, and molecular phylogenetic studies have not yet been undertaken for the genus. Based on its exceptionally large corollas, this new species does not closely resemble any of the three other species occurring in northern Mexico. *Chiococca henricksonii* M.C.Johnst. is a distinctive, narrowly endemic microphyllous species from Coahuila having tiny leaves with petioles 1–2 mm long, blades 4–9 mm long, and small solitary flowers with corollas only 7 mm long. *Chiococca petrina* Wiggins ranges from central Sonora to Chihuahua and Sinaloa. It also has relatively small puberulent leaves with petioles 1–2 mm long, blades 0.6–2 cm long, and much smaller, externally puberulent corollas 4–6 mm long with lobes about half as long as the tube. The widespread and variable *Chiococca alba* reaches its northernmost range in southern Florida, the southern tip of Texas, and also occurs in the Río Mayo region of Sonora. It differs in having inflorescences with more numerous flowers borne on a longer floral rachis and much smaller, externally glabrous corollas 6–8 mm long. Leaf size is extremely variable with petioles 3–10 mm long and blades 1.3–13 cm long, usually glabrous except the margin sometimes minutely hirtellous. Considering its morphological variability, *C. alba* may be most closely related to *C. grandiflora*.

#### Habitat and Ecology.

[Description by George M. Ferguson based on field notes and collections made at Tepopa, Sonora on 18 March 1992 and 16–17 March 1993 with Mark Fishbein, and from El Palmito, Sinaloa 12–13 April 1999 with Andy Sanders. The El Palmito site was visited by T. Van Devender in October 2017.] *Chiococca grandiflora* occurs in strikingly similar habitats at two known localities, despite a 500 km latitudinal distance apart on the Pacific versant of the Sierra Madre Occidental (Figure 3). Both are at the lower edge of pine-oak forest within a mountainous country of thick ignimbrite deposits of rhyolite and volcanic ash. Deep barranca canyons support a diversity of vegetation here where tropical and temperate zones intermix in a region subject to substantial rainy seasons in summer and winter, interspersed by marked dry seasons especially in late spring.

Near the paratype locality at 2 miles northwest of El Palmito in Sinaloa is Rancho Liebre (23.58, -105.85), located at ca. 2100 m elevation (Figure 4), a well visited birding spot on the north side of Mexico Highway 40, where one can find the narrowly endemic tufted jay or “urraca pinta” (*Cyanocorax dickey* Moore) discovered in 1934 (Crossin 1967). The area lies within the Madrean-Tropical subregion of the Madrean Ecoregion (González-Elizondo et al. 2013) which was originally defined as a Mixed Boreal-Tropical faunal region (Webb 1984). The vegetation is pine-oak forest containing elements of southern affinity with the dominant trees being *Pinus devoniana* Lindl., *P. herrerae* Martínez, *P. lumholtzii* B.L.Rob. & Fernald, *P. yecorensis* Debreczy & I.Rácz, *Quercus castanea* Née, *Q. jonesii* Trel., *Q. gentryi* C.H.Mull., *Q. mcvaughii* Spellenb., *Q. scytophylla* Liebm., *Q. subspathulata* Trel., and *Q. viminea* Trel. Other overstory trees are *Arbutus glandulosa* M.Martens & Galeotti, *A. xalapensis* Kunth, *Alnus* Mill., *Oreopanax* Decne. & Planch., and *Vachellia pennatula* (Schtdl. & Cham.) Seigler & Ebinger, and a diverse understory of shrubs, ferns and epiphytes includes *Bouvardia* Salisb., *Cuphea* P.Browne, *Garrya* Douglas ex Lindl., *Mitracarpus rhadinophyllus* (B.L.Rob.) L.O.Williams, *Oncidium* Sw., *Peperomia* Ruíz & Pavón, *Pteridium* Gled. ex Scop., *Rhus* Tourn. ex L., *Rubus* Tourn. ex L., and *Woodwardia* Sm.

The paratype localities near El Palmito are along a ridge with a divide on the rim of a spectacular, immense barranca system draining toward the northwest and another to the south. The north-facing slope of the barranca heading here has a resemblance to a cloud forest with dominant trees being *Abies durangensis* Martínez, *Quercus laurina* Bonpl., *Q. mcvaughii*, *Q. rugosa* Née, *Cornus disciflora* Moc. & Sessé ex DC., *Magnolia schiedeana* Schltdl., *Ostrya virginiana* K.Koch, *Prunus serotina* Ehrh., *Tilia mexicana* Schltdl., with species of *Clethra* Gronov. ex L., *Cercocarpus* Kunth, *Cinnamomum* Schaeffer, and *Ilex* L. At the old Rancho Liebre is an orchard and other ornamental plantings such as *Sambucus nigra* L., *Hesperocyparis* Bartel & Price, *Iris* Tourn. ex L., and *Rosa* Tourn. ex L. At the most southern paratype locality 19 km west of El Palmito, which is near the Tropic of Cancer at 1890 m along Mexico Highway 40, the vegetation here (23.466, -105.831) is lower pine-oak forest with *Pinus oocarpa* Schiede ex Schltdl. and *Pinus yecorensis* as the dominant pines and near the lower limit of *Pinus herrerae* with an abundance of oaks including *Quercus macvaughii, Q. obtusata* Bonpl., and *Q. tarahumara* Spellenb., J.D.Bacon & Breedlove, with *Arbutus xalapensis*, and *Arctostaphylos pungens* Kunth.

The more northern holotype locality for *Chicocca grandiflora* is at Tepopa, Sonora at 1100–1400 m, which is ca. 500–1000 m lower in elevation than the paratype localities (Figure 5). It is also situated in a steep northwestward draining barranca, at the ecotone of upper tropical deciduous forest including riparian evergreen forest, and lower edge of pine-oak forest with *Pinus oocarpa* and *Quercus tarahumara* with *Dodonaea viscosa* Jacq. The orchard site of Tepopa (27.3261, -108.7316) is an old ranch homestead perched above an intermittent stream at 1100 m elevation. The type specimen was probably collected along a trail between this site and the forested mesa top at 1400 m (Figure 5). From San Bernardo, Sonora into the Sierra Madre was a trail route for mule cargo that passed by here until early in the last century. Howard Scott Gentry visited this site in 1935–36 and made plant collections. An orchard, planted on a steep slope with rock lined terraces around seeps, was abandoned when Gentry visited. Paul Martin and students from the University of Arizona revisited this famous Gentry collection locale in 1992–93 (Martin et al. 1998) and found growing in the abandoned orchard avocado, banana, grapefruit, mango, orange, peach and pomegranate trees, and cultivars of native *Casimiroa edulis* La Llave and *Tecoma stans* (L.) Juss. ex Kunth.

The vegetation at Tepopa is transitional oak woodland above the uppermost tropical deciduous forest on dry slopes. Oak Woodland (with a few scattered *Pinus oocarpa* on north-facing slopes) occurs at ca. 1000–1200 m with the dominant trees being *Quercus oblongifolia* Torr. (often with an epiphytic orchid, *Laelia eyermaniana* Rchb.f.), *Q. tarahumara*, *Q. chihuahuensis* Trel., *Q. viminea* Trel., *Lysiloma watsonii* Rose, and Ipomoea arborescens (Humb. & Bonpl. ex Willd.) G.Don var. pachylutea Gentry. Understory shrubs include *Bouvardia, Cuphea, Dodonaea viscosa*, *Rhus terebinthifolia* Schltdl. & Cham., *Rubus, Tithonia calva* Sch. Bip., and the succulents *Agave bovicornuta* Gentry, *Dasylirion gentryi* Bogler, and *Ferocactus pottsii* (Salm-Dyck) Backeb.

Intergrading with oak woodland on the north-facing slopes just above Tepopa at 1200 m and upward to the mesa top at 1400 m is pine-oak forest of *Pinus oocarpa*, *P. yecorensis*, *Quercus epileuca* Trel. (with *Encyclia* Hook. orchids), *Berberis* Tourn. ex L., *Clethra, Heliocarpus* L., *Prunus serotina* Ehrh. with an understory of *Gaultheria odorata* Bredem. ex Willd., *Nolina microcarpa* S.Watson, and *Roldana hartwegii* (Benth.) H.Rob. & Brettell. Along the streambed from 900 to 1250 m is a northern extent of tropical evergreen riparian forest consisting of *Aphananthe monoica* (Hemsl.) J.-F.Leroy, *Brahea* Mart. ex Endl., *Cinnamomum hartmannii* (I.M.Johnst.) Kosterm., *Cornus disciflora*, *Ficus* Tourn. ex L., *Ilex rubra* S.Watson, *Oreopanax peltatus* Linden ex Regel, *Persea podadenia* S. F. Blake, *Prunus zinggii* Standl., *Piper villiramulum* C.DC., *Quercus tuberculata* Liebm., *Sideroxylon tepicense* (Standl.) T.D.Penn., *Urera* Gaudich., epiphytic *Tillandsia cretacea* L.B.Sm. and a large orchid, *Stanhopea maculosa* Knowles & Westc., at its northern-most distribution.

#### Proposed Conservation Status.

Endangered: EN B1ab(iii) + 2ab (iii). It is possible that *Chiococca grandiflora* is more widespread and/or abundant than the small number of collections suggests, since areas between and around the known sites in Sinaloa and Sonora have not been well explored botanically. However, based on the best available evidence this species falls into the IUCN Red List Criteria (IUCN 2001) category of Endangered (EN): B1: total Extent of Occurrence (EOO) is less than 5,000 km^2^ (ca. 495 km^2^); B1a, is severely fragmented and known to exist at no more than five locations; B1biii, continuing decline inferred in area, extent and/or quality of habitat; B2: total area of occupancy (AOO) less than 500 km^2^ (ca. 12 km^2^), B2a, severely fragmented or known to exist at no more than five locations, and B2b iii, continuing decline inferred in area, extent and/or quality of habitat. The suitable habitat for *Chiococca grandiflora* is a declining or endangered environment threatened by human activity (deforestation, fire).

## Supplementary Material

XML Treatment for Chiococca grandiflora
